# Glaucoma Diagnostic Accuracy of Machine Learning Classifiers Using Retinal Nerve Fiber Layer and Optic Nerve Data from SD-OCT

**DOI:** 10.1155/2013/789129

**Published:** 2013-11-28

**Authors:** Kleyton Arlindo Barella, Vital Paulino Costa, Vanessa Gonçalves Vidotti, Fabrício Reis Silva, Marcelo Dias, Edson Satoshi Gomi

**Affiliations:** ^1^Faculty of Medical Sciences, Universidade Estadual de Campinas (UNICAMP), Campinas, SP, Brazil; ^2^Department of Engineering, University of São Paulo (USP), São Paulo, SP, Brazil

## Abstract

*Purpose*. To investigate the diagnostic accuracy of machine learning classifiers (MLCs) using retinal nerve fiber layer (RNFL) and optic nerve (ON) parameters obtained with spectral domain optical coherence tomography (SD-OCT). *Methods*. Fifty-seven patients with early to moderate primary open angle glaucoma and 46 healthy patients were recruited. All 103 patients underwent a complete ophthalmological examination, achromatic standard automated perimetry, and imaging with SD-OCT. Receiver operating characteristic (ROC) curves were built for RNFL and ON parameters. Ten MLCs were tested. Areas under ROC curves (aROCs) obtained for each SD-OCT parameter and MLC were compared. *Results*. The mean age was 56.5 ± 8.9 years for healthy individuals and 59.9 ± 9.0 years for glaucoma patients (*P* = 0.054). Mean deviation values were −1.4 dB for healthy individuals and −4.0 dB for glaucoma patients (*P* < 0.001). SD-OCT parameters with the greatest aROCs were cup/disc area ratio (0.846) and average cup/disc (0.843). aROCs obtained with classifiers varied from 0.687 (CTREE) to 0.877 (RAN). The aROC obtained with RAN (0.877) was not significantly different from the aROC obtained with the best single SD-OCT parameter (0.846) (*P* = 0.542). *Conclusion*. MLCs showed good accuracy but did not improve the sensitivity and specificity of SD-OCT for the diagnosis of glaucoma.

## 1. Introduction

Primary open-angle glaucoma is a chronic disease that is characterized by a progressive optic neuropathy and degeneration of the retinal nerve fiber layer (RNFL), resulting in a distinct appearance of the optic nerve head (ONH) and concomitant visual field (VF) loss. Examination of the RNFL and the ONH are recognized as valuable methods of diagnosing early glaucoma, since these changes are often detectable before VF loss [1]. Some studies have shown that as many as half of retinal ganglion cells can be lost before standard automated perimetry (SAP) shows a VF defect [2, 3]. During the last years, several methods have emerged for the objective assessment of RNFL thickness and ONH topography [4].

Optical coherence tomography (OCT), first described by Huang et al. in 1991 [5], has been widely accepted in glaucoma management [6]. The Cirrus spectral domain OCT (SD-OCT) (Carl Zeiss Meditec Inc., Dublin, CA), one of the commercially available SD-OCT instruments, has an axial resolution of 5 *μ*m and a scan speed of 27,000 A-scans per second. The scanning area covers 6 mm × 6 mm × 2 mm, analyzing both RNFL thickness and ONH topography. This SD-OCT provides faster scanning than previous time domain OCTs (TD-OCT) [7].

Machine learning classifiers (MLCs) have been developed since 1962 [8] and have been used in ophthalmology research since 1990 [9]. MLCs train computerized systems to detect the relationship between multiple input parameters, eventually facilitating the diagnosis of a condition. In fact, some reports suggest that MLCs are as good as [10–13] or better than [14–20] currently available techniques for glaucoma diagnosis.

In a recent study [21], we have demonstrated that MLCs using RNFL thickness measurements obtained with SD-OCT show good diagnostic accuracy. However, they did not improve the sensitivity and specificity of RNFL parameters alone. In a subsequent study, we analyzed the accuracy of MLCs using RNFL and VF parameters [22]. The purpose of this study is to evaluate the sensitivity and specificity of MLCs using both RNFL and ONH parameters measured by SD-OCT for the diagnosis of glaucoma.

## 2. Methods

### 2.1. Subjects

This was a prospective, observational, cross-sectional study. We analyzed 103 eyes of 103 participants (46 healthy control subjects and 57 patients with glaucoma), all of them older than 40 years, at the Glaucoma Service of the University of Campinas (UNICAMP), Brazil. Each participant had a complete ophthalmic evaluation that included medical history, best corrected visual acuity (BCVA), slit lamp biomicroscopy, measurement of intraocular pressure (IOP) with Goldmann tonometry, gonioscopy, dilated slit lamp fundus examination with a 78-diopter lens, SAP using the standard 24-2 Swedish interactive threshold algorithm (SITA) (Humphrey Field Analyzer II, Carl Zeiss Meditec Inc., Dublin, CA), and imaging with the Cirrus SD-OCT. All patients participated in two other studies published previously by our group [21, 22]. However, after an upgrade of the Cirrus software (5.1.1.6), which allows the analysis of the ONH, a change in the signal strength of almost all OCT images was observed. We decided to modify the inclusion criteria, decreasing the minimum signal strength to 6 (instead of 7), which resulted in a total of 103 eyes of 110 participants.

Participants of both groups had a BCVA better or equal to 20/40, spherical refraction within ±5.0 diopters (D), cylinder correction within ±3.0 D and open angles on gonioscopy and reliable SAPs with false-positive errors <33%, false-negative errors <33%, and fixation losses <20%. We excluded all eyes with retinal diseases, uveitis, pseudophakia or aphakia, nonglaucomatous optic neuropathy, and significant cataract according to the criteria of Lens Opacification Classification System III (LOCSIII) [23], defined as the maximum nuclear opacity (NC3, NO3), cortical (C3), and subcapsular (P3). If both eyes were eligible, one eye was randomly selected.

The inclusion criteria for healthy eyes were IOP ≤ 21 mmHg with no history of elevated IOP or glaucoma cases in the family and two consecutive and reliable normal visual 3 fields.

The inclusion criteria for glaucomatous eyes were two or more IOP measurements >21 mmHg and a glaucomatous VF defect confirmed in two recent and reliable examinations. Eyes with glaucomatous VF defects were defined as those that met two of the following criteria: (1) cluster of 3 points with a probability of <5% on a pattern deviation map in a single hemifield, including at least 1 point with a probability of <1%; (2) glaucoma hemifield test outside 99% of the age-specific normal limits; and (3) pattern standard deviation outside 95% of the normal limit. The severity of glaucomatous damage was classified into (a) mild damage: mean deviation (MD) ≥ −6 dB; (b) moderate damage: MD between −6 dB and −15 dB; (c) advanced damage: MD ≤ −15 dB. Glaucomatous eyes with advanced damage were excluded from this study.

We respected the Declaration of Helsinki and obtained an informed consent from all participants. The study was approved by the University of Campinas Medical Institutional Review Board.

### 2.2. Optical Coherence Tomography

All subjects had RNFL thickness and ONH topography measured with the Cirrus SD-OCT (software version 5.1.1.6). The ONH mode consists in a 3-dimensional dataset of 200 A-scans that are derived from 200 B-scans and analyzes a 6-mm^2^ area centered on the optic disc. The software creates a RNFL thickness map from the 3-dimensional cube data set and centers the disc. Subsequently, it also extracts a circumpapillary circle of 1.73 mm of radius for RNFL thickness measurements. The SD-OCT provides RNFL thickness maps with 4 quadrants (superior, inferior, nasal, and temporal) and 12-clock-hours and average thickness measurements. All RNFL hour measurements were aligned according to the orientation of the right eye. Hence, clock hour 3 of the circumpapillary scan represented the nasal side of the optic disc for both eyes. The 5.1.1.6 software also allows the measurement of ONH parameters, such as rim area, disc area, average cup/disc ratio, vertical cup/disc ratio and cup volume. We created an additional parameter: the cup/disc area ratio, defined as: [(disc area − rim area)/disc area]. The end of Bruch's membrane is defined as the disc margin and is identified from the 3-dimensional cube dataset. The rim width around the circumference of the optic disc edge is determined by measuring the amount of neuroretinal tissue in the optic nerve [6]. We excluded all poor-quality scans analyzed at printouts with (a) incorrect identification of the vitreoretinal surface, (b) horizontal eye motion within the measurement circle in the *en face* image printouts, and (c) misidentification of Bruch's membrane. Only well-centered scans with a signal strength between 6 and 10 were included. All images were acquired with undilated pupils by a single, well-trained ophthalmologist, masked for the diagnosis.

### 2.3. Machine Learning Classifiers

Ten MLC algorithms were tested using 23 parameters measured with the SD-OCT (17 RNFL and 6 ONH). The following MLCs were tested: bagging (BAG), naïve-bayes (NB), linear support vector machine (SVML), Gaussian support vector machine (SVMG), multilayer perceptron (MLP), radial basis function (RBF), random forest (RAN), ensemble selections (ENS), classification tree (CTREE), and AdaBoost M1 (ADA). The rationale behind each MLC was explained in a previous paper [21]. Initially, the classifiers were trained with all 23 SD-OCT parameters. Then, a backward feature selection was used to find the smallest number of parameters that resulted in the best accuracy. The analysis started with the full-dimensional feature set and sequentially deleted the feature with worst accuracy (based on the aROC) and restarted a new analysis.

Weka software version 3.7.7 (Waikato Environment for Knowledge Analysis, the University of Waikato, New Zealand) was used to develop all 10 classifiers. Both receiver operating characteristic (ROC) curves and the calculation of the area under the ROC curve (aROC) were obtained using this software.

We used the 10-fold cross-validation resampling method to maximize the use of our data. All eyes were randomly divided into 10 subsets, each containing approximately the same number of healthy and glaucomatous eyes. Nine subsets were used for training the classifiers, while the remaining subset was used for testing the classification performance.

### 2.4. Statistical Analysis

MedCalc software version 12.3.0 (MedCalc Software, Mariakerke, Belgium) was used in all analysis. Continuous variables were compared using the Student's *t*-test and categorical variables were analyzed using the chi-square test.

aROCs were obtained for all 23 SD-OCT parameters: average thickness, 4 quadrants (superior, inferior, nasal, and temporal), and 12-clock-hours RNFL thickness measurements, rim area, disc area, cup/disc area, average cup/disc, vertical cup/disc, and cup volume measurements. aROCs obtained for each SD-OCT parameter and each machine learning classifier, before and after optimization, were compared using the *z* test. *P* values <0.05 were considered to be statistically significant.

## 3. Results

One hundred and three eyes of 103 patients were enrolled in this study; 46 of them were healthy eyes and 57 glaucomatous eyes.

The clinical characteristics of the study population are shown in Table 1. The mean age was 56.5 ± 8.9 years for healthy individuals and 59.9 ± 9.0 years for glaucoma patients (*P* = 0.054). There was no significant difference between groups regarding IOP (14.7 ± 2.6 mmHg and 13.8 ± 2.5 mmHg, resp.) (*P* = 0.100), but glaucoma patients were using a mean number of 2.0 ± 1.1 medications to lower IOP. Mean MD values were −1.4 ± 1.6 dB for healthy individuals and −4.0 ± 2.4 dB for glaucoma patients (*P* < 0.001). Among the glaucoma patients, 86.0% had early VF damage and 14.0% had moderate VF damage.

Table 2 compares the mean SD-OCT values in both groups. All SD-OCT parameters were significantly different between the groups, except for the 3, 4, and 9 o'clock positions and disc area.

Table 3 displays the aROCs of all SD-OCT parameters. The parameters with larger aROCs with a 95% confidence interval (CI) were cup/disc area (0.846—CI 0.762–0.910), average cup/disc (0.843—CI 0.758–0.907), vertical cup/disc (0.832—CI 0.746–0.899), rim area (0.828—CI 0.741–0.895), cup volume (0.786—CI 0.694–0.860), average thickness (0.783—CI 0.690–0.858), and inferior (0.775—CI 0.682–0.851). For a fixed specificity of 80%, the best sensitivities were observed with vertical cup/disc (70.8%), rim area (70.1%), cup/disc area (67.7%), average cup/disc (66.6%), cup volume (64.9%), and inferior (63.1%). For a fixed specificity of 90%, the best sensitivities were observed with rim area (62.4%), cup/disc area (60.0%), vertical cup/disc (58.9%), average cup/disc (58.2%), superior (55.4%), and average thickness (51.9%).

**Figure 1:**
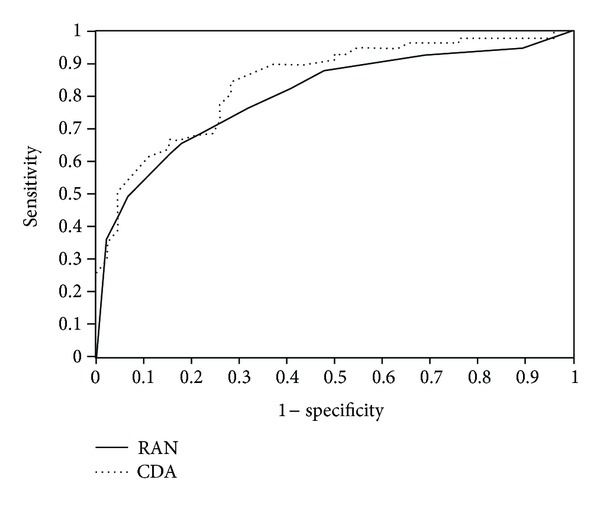
Areas under the receiver operating characteristic curve (aROCs) of the best classifier trained with the number of spectral domain optical coherence tomography (SD-OCT) parameters which allowed the best performance (RAN: random forest = 0.877) and aROC of the best SD-OCT parameter (CDA: cup/disc area = 0.846) (*P* = 0.542).

aROCs obtained with MLCs varied from 0.687 (CTREE) to 0.839 (ADA and RBF) when trained with all parameters. Nine classifiers performed best when trained with a smaller number of parameters. When less parameters were used, aROCs varied from 0.733 (CTREE) to 0.877 (RAN) (Table 4). The best aROC obtained with RAN trained with 13 parameters (0.877) was not significantly different from the aROC obtained with the best single optic nerve SD-OCT parameter (cup/disc area) aROC = 0.846 (*P* = 0.542) (Figure 1) and from the aROC obtained with the best single retinal nerve fiber layer SD-OCT parameter (average thickness) aROC = 0.783 (*P* = 0.094).

When a small number of parameters was used, there was an increase in the aROCs of 9 of 10 classifiers: SVML (13.0% increase), MLP (9.7%), SVMG (9.5%), RAN (8.9%), CTREE (6.6%), NB (6.3%), ENS (4.5%), RBF (3.2%) BAG (2.9%), and ADA (0%). However, this increase was not statistically significant (*P* > 0.05).

## 4. Discussion

Statistical comparisons between the various published studies that investigate the glaucoma diagnostic accuracy are difficult because of different demographic distributions, inclusion and exclusion criteria, OCTs and MLCs employed and, mainly, the severity of glaucoma.

Since its inception, MLCs have been studied in combination with several apparatus designed to improve the diagnosis of glaucoma such as TD-OCT [11, 15, 17, 19], SD-OCT [21, 22], Heidelberg Retina Tomograph (HRT) [16, 18, 24], Scanning Laser Polarimetry (GDx) [14], and VF [12, 17, 18, 20, 22].

In the scientific literature, we identified six relevant studies involving the structural analysis of the ONH with OCT and/or HRT associated with MLCs in order to improve the accuracy in the diagnosis of glaucoma. Similar to previous reports, our study demonstrated that the reduction in the number of OCT parameters improved the performance of MLC [10, 11, 16, 19, 21, 22, 24]. However, there is disagreement about the superiority of MLCs over isolated parameters for the diagnosis of glaucoma.

As far as we know, this study was the first to use MLCs with both RNFL and ONH data obtained from SD-OCT trying to improve the glaucoma diagnostic accuracy. We found that the best classifier was RAN (aROC = 0.877) and the best individual parameter from SD-OCT was the cup/disc area ratio (aROC = 0.846), with no statistical difference between them (*P* = 0.542). We consider those results as reflecting a good diagnostic accuracy, especially because 86% of the glaucomatous eyes were classified as having mild VF damage (MD > −6 dB).

Burgansky-Eliash et al. used five MLCs built with RNFL, ONH, and macular data from TD-OCT (a total of 38 parameters, of which only the 8 parameters with the best correlation with MD were used). They examined 42 healthy eyes and 47 glaucomatous eyes (among these, 27 eyes with early glaucoma and 20 eyes with advanced glaucoma). The healthy subjects were significantly younger than the glaucoma patients (*P* = 0.001) and the mean VF MD of the glaucomatous eyes was −6.4 dB. They concluded that the aROC obtained with the best classifier SVM (0.981) was not significantly different from the aROC obtained with the best single ONH parameter of OCT, the rim area (0.969) (*P* = 0.07). On the other hand, the aROC obtained with SVM was significantly larger than the best single RNFL parameter average thickness (0.938) and mean macular thickness (0.839) (*P* = 0.01 and *P* < 0.001, resp.) [11]. In our study, we did not observe differences between aROCs obtained with RNFL parameters compared with those obtained with classifiers. However, the previous study used VF information to reduce the number of OCT parameters, which could have introduced bias, adding functional information to a classifier that should have exclusively structural data.

In a study of our group, Vidotti et al. compared the performance of 17 RNFL parameters from SD-OCT and 10 MLCs in discriminating between 48 healthy and 62 glaucomatous eyes. The best individual parameter was inferior quadrant (aROC = 0.813), the best classifier trained with all OCT parameters was SVMg (aROC = 0.795), and the best classifier trained with two SD-OCT parameters was BAG (aROC = 0.818) (*P* = 0.93) [21]. Similar to our study, a large proportion (82.3%) of their patients had early glaucomatous VF damage. Both studies did not find any significant improvement in discriminating patients with glaucoma from healthy subjects comparing the best single OCT parameter and the best MLC.

On the other hand, Bizios et al. tested the performance of two MLCs (MLP and SVM) using conventional and new RNFL thickness data from TD-OCT. The authors analyzed 90 healthy subjects and 62 glaucomatous eyes with MD > −12 dB, combined with changes in optic nerve photographs. Both MLCs (MLP and SVM) performed successfully with aROCs of 0.982 (95% CI 0.966–0.999) and 0.989 (95% CI 0.979–1), respectively. SVM trained on the transformed A-scan thickness values performed significantly better than MLPs or SVMs trained on any of the single RNFL thickness parameters (*P* = 0.038). SVM performance based on this input was also better than the performance of the average RNFL thickness (*P* = 0.013) [15]. Likewise, Huang et al. tested three MLCs in order to improve the accuracy of glaucoma diagnosis based on RNFL and ONH data obtained with TD-OCT. They analyzed 100 normal individuals and 89 glaucomatous patients with early VF damage (MD > −6 dB). The inferior quadrant thickness was the best individual OCT parameter (aROC = 0.832) and Mahalanobis was the best MLC (aROC = 0.849). However, there is no statistical comparison between the aROCs obtained with inferior quadrant and Mahalanobis in this paper [19].

Naithani et al. evaluated the relationship between RNFL and ONH from TD-OCT and HRTII and compared three TD-OCT-based MLCs with those inbuilt in HRTII for detection of glaucomatous damage. As we know, OCT and HRT evaluate the ONH with two different scanning techniques. Furthermore, they use distinct reference planes to define where the cup begins, which may cause a difference in the measured values of all ONH parameters evaluated by the two modalities. They evaluated 60 normal eyes and 60 glaucomatous eyes, 30 of those with early glaucoma and 30 with moderate glaucoma. LDA was the best MLC-OCT parameter (aROC = 0.982) and FSM functions as the best MLC-HRT parameter (aROC = 0.859). Although there was no statistical comparison between those values, they concluded that OCT algorithms perform better than HRT-based formulas in distinguishing patients with early or moderate glaucoma from normal subjects [24].

Finally, Townsend et al. aimed to assess the performance of seven classifiers trained on HRTIII parameters for discriminating between 60 healthy eyes and 140 glaucomatous eyes. The classifiers were trained on all 95 variables and smaller sets created with backward elimination. The aROC was calculated for classifiers, individual parameters, and HRTIII glaucoma probability scores (GPS). Vertical cup/disc ratio was the individual parameter with the best performance (aROC = 0.848), global GPS was the best GPS parameter (aROC = 0.829), and SVMr showed significant improvement over both (aROC = 0.904) (*P* = 0.018 and *P* = 0.006, resp.). They concluded that MLC can provide a significant improvement in HRTIII diagnostic power over single parameters and GPS [16].

Our study has limitations, including a limited sample size in both groups and the use of 10-fold cross-validation resampling method that maximizes the analysis of our data but uses the same population to train MLCs and test their performance.

In conclusion, the MLCs obtained with RNFL and ONH data did not improve the sensitivity and specificity of the Cirrus SD-OCT for the diagnosis of mild to moderate glaucoma in this population, even though a good diagnostic accuracy was observed. Further studies with a larger sample, pool of new structural parameters of OCT, and new classifiers may improve the accuracy for the diagnosis of glaucoma.

## Figures and Tables

**Table 1:** Demographic characteristics of the healthy and glaucoma groups.

	Healthy (*n* = 46)	Glaucoma (*n* = 57)	*P*
Age (years; mean ± SD)	56.5 ± 8.9	59.9 ± 9.0	0.054
Gender (male [%] : female [%])	23 [50.0] : 23 [50.0]	28 [49.2] : 29 [50.8]	0.930
Race (Caucasian [%] : African-American [%])	35 [76.0] : 11 [24.0]	43 [75.4] : 14 [24.6]	0.539
Visual acuity LogMAR (mean ± SD)	0.04 ± 0.09	0.09 ± 0.10	**0.010**
Spherical equivalent (diopters; mean ± SD)	0.78 ± 1.6	0.95 ± 1.4	0.586
Intraocular pressure (mmHg; mean ± SD)	14.7 ± 2.6	13.8 ± 2.5	0.100
Medications (mean ± SD)	0	2.0 ± 1.1	**<0.001**
MD (dB; mean ± SD)	−1.4 ± 1.6	−4.0 ± 2.4	**<0.001**
PSD (dB; mean ± SD)	1.8 ± 0.7	4.3 ± 2.4	**<0.001**

SD: standard deviation; MD: mean deviation; dB: decibel; PSD: pattern standard deviation.

**Table 2:** Mean ± standard deviation of SD-OCT parameters in both groups.

SD-OCT	Healthy (*n* = 46)	Glaucoma (*n* = 57)	*P*
Average thickness (*µ*m)	93.3 ± 9.9	81.4 ± 11.2	**<0.001**
Quadrant (*µ*m)			
Temporal	63.2 ± 11.3	57.4 ± 12.5	**0.017**
Superior	117.4 ± 15.1	100.4 ± 18.9	**<0.001**
Nasal	72.8 ± 10.9	66.4 ± 9.8	**0.002**
Inferior	119.9 ± 17.6	101.1 ± 17.5	**<0.001**
Clock hour (*µ*m)			
1	103.3 ± 19.0	90.9 ± 20.8	**0.002**
2	90.9 ± 16.2	78.7 ± 13.8	**<0.001**
3	61.4 ± 9.3	59.4 ± 9.5	0.295
4	66.2 ± 13.7	62.1 ± 11.7	0.105
5	97.2 ± 18.9	88.0 ± 15.6	**0.007**
6	132.4 ± 26.4	111.1 ± 25.3	**<0.001**
7	130.2 ± 23.4	104.3 ± 27.8	**<0.001**
8	64.7 ± 13.5	58.4 ± 15.7	**0.033**
9	50.8 ± 14.5	48.1 ± 12.8	0.316
10	74.2 ± 13.6	65.9 ± 14.4	**0.003**
11	123.8 ± 21.9	103.2 ± 26.7	**<0.001**
12	125.0 ± 26.1	107.0 ± 25.6	**<0.001**
Cup/disc area (ratio)	0.34 ± 0.14	0.53 ± 0.13	**<0.001**
Average cup/disc (ratio)	0.56 ± 0.13	0.71 ± 0.09	**<0.001**
Vertical cup/disc (ratio)	0.53 ± 0.13	0.68 ± 0.09	**<0.001**
Rim area (mm^2^)	1.28 ± 0.21	0.98 ± 0.24	**<0.001**
Cup volume (mm^3^)	0.24 ± 0.24	0.50 ± 0.29	**<0.001**
Disc area (mm^2^)	2.01 ± 0.41	2.19 ± 0.52	0.055

SD-OCT: spectral domain optical coherence tomography.

**Table 3:** Areas under the ROC curve (aROCs) for each SD-OCT parameter and sensitivities (%) with fixed specificities of 80% and 90%.

SD-OCT	aROC (CI)	Specificity 80%	Specificity 90%
Average thickness	0.783 (0.690–0.858)	62.2	51.9
Quadrant			
Temporal	0.641 (0.540–0.733)	38.7	28.0
Superior	0.747 (0.652–0.828)	57.8	55.4
Nasal	0.672 (0.573–0.761)	41.9	23.8
Inferior	0.775 (0.682–0.851)	63.1	45.6
Clock hour			
1	0.690 (0.591–0.777)	49.1	27.3
2	0.720 (0.623–0.804)	52.9	45.9
3	0.563 (0.462–0.661)*	23.1	18.4
4	0.597 (0.495–0.692)*	26.4	12.2
5	0.642 (0.542–0.734)	28.0	25.6
6	0.711 (0.613–0.796)	45.6	32.6
7	0.764 (0.670–0.842)	54.7	40.7
8	0.638 (0.537–0.730)	44.2	26.6
9	0.564 (0.463–0.662)*	31.5	25.7
10	0.670 (0.570–0.759)	47.7	31.9
11	0.741 (0.646–0.823)	58.6	32.6
12	0.686 (0.587–0.774)	42.8	24.5
Cup/disc area	0.846 (0.762–0.910)	67.7	60.0
Average cup/disc	0.843 (0.758–0.907)	66.6	58.2
Vertical cup/disc	0.832 (0.746–0.899)	70.8	58.9
Rim area	0.828 (0.741–0.895)	70.1	62.4
Cup volume	0.786 (0.694–0.860)	64.9	42.1
Disc area	0.594 (0.493–0.690)*	33.3	19.3

SD-OCT: spectral domain optical coherence tomography; CI: confidence interval of 95%.

*Parameters with aROCs not significantly different from chance.

**Table 4:** Areas under the receiver operating characteristic curve (aROCs) of best parameter (BP) and all 23 parameters (AP) obtained with machine learning classifiers and sensitivities (%) with fixed specificities of 80% and 90% for AP.

MLC	aROC-BP (CI) [NP]	aROC-AP (CI)	Specificity 80%-AP	Specificity 90%-AP
RAN	0.877 (0.810–0.944) [13]	0.805 (0.738–0.872)	64.9	49.1
NB	0.870 (0.801–0.939) [11]	0.818 (0.749–0.939)	68.4	52.6
RBF	0.866 (0.796–0.936) [11]	0.839 (0.746–0.898)	71.9	63.1
MLP	0.843 (0.768–0.918) [11]	0.768 (0.693–0.918)	49.1	47.3
ADA	0.839 (0.763–0.915) [19]	0.839 (0.763–0.915)	73.6	52.6
ENS	0.829 (0.751–0.907) [08]	0.793 (0.715–0.871)	61.4	56.1
BAG	0.828 (0.749–0.907) [12]	0.804 (0.725–0.883)	57.8	50.8
SVMG	0.825 (0.746–0.904) [10]	0.753 (0.674–0.832)	56.0	28.0
SVML	0.780 (0.692–0.868) [02]	0.690 (0.602–0.778)	45.0	22.5
CTREE	0.733 (0.684–0.862) [07]	0.687 (0.638–0.736)	46.0	23.0

MLC: machine learning classifier; aROC: area under the ROC curve; BP: best parameter; AP: all parameters; NP: number of parameters; CI: confidence interval of 95%; BAG: bagging; NB: Naive-Bayes; SVML: linear support vector machine; SVMG: Gaussian support vector machine; MLP: multilayer perceptrons; RBF: radial basis function; RAN: random forest; ENS: ensemble selection; CTREE: classification trees; ADA: AdaBoost.
